# Lower Sphingomyelin SM 42:1 Plasma Level in Coronary Artery Disease—Preliminary Study

**DOI:** 10.3390/ijms26041715

**Published:** 2025-02-17

**Authors:** Tomasz Urbanowicz, Paweł Gutaj, Szymon Plewa, Ievgen Spasenenko, Beata Krasińska, Anna Olasińska-Wiśniewska, Dariusz Kowalczyk, Zbigniew Krasiński, Ewelina Grywalska, Mansur Rahnama-Hezavah, Mariusz Kowalewski, Andrzej Tykarski, Ewa Wender-Ożegowska, Jan Matysiak

**Affiliations:** 1 Cardiac Surgery and Transplantology Department, Poznan University of Medical Sciences, 61-848 Poznan, Poland; 2 Thoracic Research Centre, Collegium Medicum Nicolaus Copernicus University, Innovative Medical Forum, 85-067 Bydgoszcz, Poland; 3 Department of Reproduction, Poznan University of Medical Sciences, 60-535 Poznan, Poland; 4 Department of Inorganic and Analytical Chemistry, Faculty of Pharmacy, Poznan University of Medical Sciences, 60-812 Poznan, Poland; 5 Department of Hypertensiology, Angiology and Internal Medicine, Poznan University of Medical Sciences, 61-848 Poznan, Poland; 6 Faculty of Health Sciences, University of Kalisz, 62-800 Kalisz, Poland; 7 Department of Vascular, Endovascular Surgery, Angiology and Phlebology Medical, Poznan University of Medical Science, 61-848 Poznan, Poland; 8 Department of Experimental Immunology, Medical University of Lublin, 20-059 Lublin, Poland; 9 Department of Dental Surgery, Medical University of Lublin, 20-059 Lublin, Poland; 10 Clinical Department of Cardiac Surgery and Transplantology, National Medical Institute of the Ministry of Interior and Administration, 02-507 Warsaw, Poland

**Keywords:** CAD, SM 42:1, sphingomyelin, Gensini, metabolomic

## Abstract

Coronary artery atherosclerosis is a common condition characterized by different symptomatology and incidences of risk factors. The disease manifestation may differ; therefore, proper diagnosis is essential. The preventive, diagnostic, and therapeutic arms are still developing to improve patient outcomes. Among diagnostic steps, the non-invasive tools for evaluating non-classical factors related to metabolomic profiles are gaining attention. The aim of this study was to investigate possible metabolic profiling differences between patients with chronic coronary artery disease (CAD) and a control group based on plasma sphingomyelin levels. The study group consisted of 23 patients (72% male, median age of 69 (63–72) years) presenting with chronic coronary syndrome and confirmed epicardial disease in coronary angiography and 15 patients (33% male, median age of 70 (64–72) years) with normal angiographic results. Clinical data were recorded, and blood samples were collected for standard biochemical laboratory assessment and metabolomic profiling. The plasma sphingomyelin levels were evaluated in patients with different degrees of coronary artery atherosclerosis involvement. In addition, the severity of the epicardial disease was estimated by the Gensini Score. The study subgroups did not differ in terms of age (*p* = 0.765) and co-morbidities, though the male sex was more common in the CAD group (*p* = 0.007). The analysis revealed significant differences regarding neutrophil count (*p* = 0.014), neutrophil-to-lymphocyte ratio (NLR) (*p* = 0.016), and high-density lipoprotein (HDL) (*p* = 0.003). Among different plasma sphingomyelin species, there was a significant difference in plasma SM42:1 level (16.2 (14.2–19.1) vs. 20.8 (18.9–21.7) (*p* = 0.044) between the CAD and control groups, respectively. The SM 42:1 plasma level was independent of the number of involved epicardial arteries (*p* = 0.109). However, Spearman correlations tests were performed between the SM 42:1 plasma level and the number of coronary arteries diagnosed with atherosclerosis disease (rho = −0.356, *p* = 0.014) and the severity of the disease measured by the Gensini Score (rho = −0.403, *p* = 0.006). There was no correlation between plasma sphingomyelin levels and NLR (Spearman’s rho = −0.135, *p* = 0.420), suggesting a lack of inflammatory associations. Further, sphingomyelins showed no relationship with coronary artery disease risk factors such as dyslipidemia and diabetes. Lower plasma SM 42:1 levels were revealed in the CAD group compared with the control group, indicating a possible significance of sphingomyelin 42:1 in coronary artery disease progression.

## 1. Introduction

Notable improvements related to atherosclerosis disease have been made to cardiovascular medicine over the past decades to improve diagnosis, control risk factors, and expand life span. This pathologic condition is linked to multivariable factors characterized by various symptoms and a long asymptomatic course [1]. Disease manifestation may differ; therefore, proper management is essential. Three main tasks are involved in managing patients with coronary artery disease (CAD)—primary and secondary prevention, diagnostics, and medical and interventional treatment.

Preventive medicine focuses on lifestyle modifications related to a healthy diet and physical activity. The concordant evidence indicates low salt consumption and increased intake of plant-based foods—whole grains, vegetables, fruits, legumes, and nuts combined with olive oil and other unsaturated-fat-rich oils [2]. Multicenter studies confirmed the beneficial role of moderate to vigorous physical activity in coronary and carotid atherosclerosis prevention [3].

Diagnostic medicine focuses on non-invasive biochemical interventions, including imaging-based biomarkers for atherosclerosis diagnosis [4,5,6]. Early biomarker identification is linked to the possible association of plasma molecules with atherosclerotic plaque susceptibility to ulceration in preventing life-threatening events [7]. In chronic conditions, improving proteomic tools and metabolomics profiling attempts to unravel pathways of atherosclerotic formation and progression [8].

Interventional medicine has changed within the last decade, offering new techniques and improving long-term results [9,10,11,12]. Surgical intervention, though associated with higher periprocedural risk, offers a long-term reduction of recurrent cardiac events [13]. The beneficial role of revascularization is to improve patients’ prognosis at long-term follow-up, even in ischemic cardiomyopathy [14].

The preventive, diagnostic, and therapeutic arms are still developing to improve patients’ outcomes. Among diagnostic steps, the non-invasive tools for evaluating non-classical factors related to metabolomic profile are gaining attention. The aim of the presented study was to investigate possible metabolic profiling differences between patients with chronic coronary artery disease (CAD group) and a control group based on plasma sphingomyelin levels.

## 2. Results

The prospective single-center analysis comprised 23 (18 (78%) males and five (22) females) white Caucasian patients with a median (Q1–Q3) age of 69 (63–72) years with proven epicardial atherosclerosis involving one (*n* = 3), two (*n* = 5), and three (*n* = 10) coronary arteries or left main disease (*n* = 5). The severity of the epicardial disease was estimated by the Gensini Score, reaching the median (Q1–Q3) value of 52 (38–67).

The control group included 15 (five (33%) male and 10 (67%) female) white Caucasian patients with a median age of 70 (64–72) years.

There were no significant differences between the groups regarding age (*p* = 0.765), arterial hypertension (*p* = 0.587), diabetes mellitus (*p* = 0.820), dyslipidemia (*p* = 0.759), chronic obstructive pulmonary disease (*p* = 0.228), kidney chronic disease (*p* = 0.555), and smoking (*p* = 0.240). The groups varied in terms of sex differences (*p* = 0.007), correlating with the natural prevalence of CAD disease in epidemiological studies [15].

The groups’ demographics and clinical characteristics are presented in Table 1.

The laboratory test results, including peripheral blood count analysis, liver and kidney function tests, and heart failure markers, were compared between the groups. Whole blood count analysis revealed significant differences regarding neutrophil count (*p* = 0.014), neutrophil-to-lymphocyte ratio (NLR) (*p* = 0.016), and red cell distribution width (RDW) (*p* = 0.048).

The lipogram analysis revealed significant differences regarding high-density lipoprotein (HDL) (*p* = 0.003) but not low-density lipoprotein (LDL) (*p* = 0.843) or triglycerides (*p* = 0.781). Neither lipoprotein (a) serum concentration (*p* = 0.067) nor uric acid (*p* = 0.126) distinguished the groups.

Though within the normal range (up to 40 ng/mL), myocardial injury markers, such as creatine kinase-MB (CK-MB), discriminated the groups (14.85 (3.03–16.02) ng/mL vs. 0.74 (0.58–1.70) ng/mL) (*p* < 0.001). The detailed information is presented in Table 2.

The metabolomic profiles associated with multiple plasma sphingomyelin levels were analyzed and compared between the groups, indicating significant differences in plasma SM42:1 level (16.2 (14.2–19.1) μM vs. 20.8 (18.9–21.7) μM, (*p* = 0.044)), as presented in Table 3.

**Figure 1:**
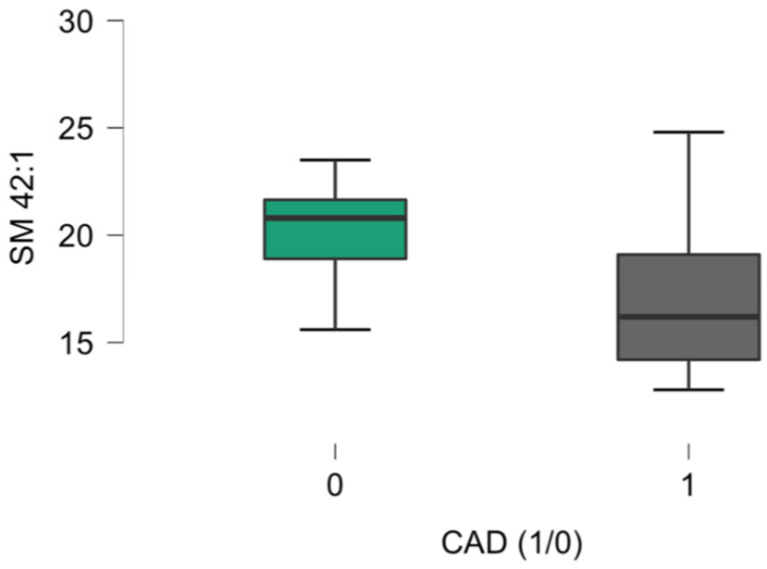
Plasma SM 42:1 level differences (*p* = 0.044) between non-CAD (0) and CAD (1) groups.

Significant differences in SM 42:1 were observed between the CAD (16.2 (14.2–19.1) μM) and non-CAD (20.8 (18.9–21.7)) μM groups, as presented in Figure 1.

Sex differences were observed between the groups, with Fisher’s exact test revealing a predictive value of neutrophil count (Log Odds ratio: 1.99, 95% CI: 1.25–2.77, *p* < 0.001) and SM 42:1 for coronary disease in male patients. The results are presented in Table 4.

**Figure 2:**
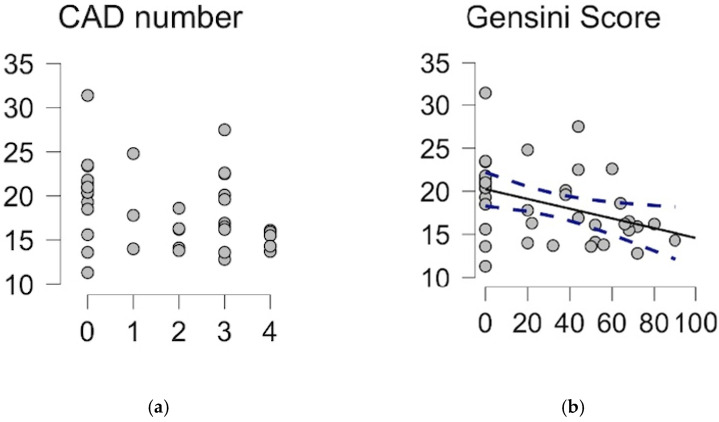
The relationship between SM 42:1 plasma level and severity of coronary artery disease as estimated by the number of involved arteries (**a**) and the Gensini Score (**b**). 0—no coronary atherosclerotic changes, 1—one-vessel disease; 2—two-vessel disease; 3—multivessel disease; 4—left main disease. Dots – particular patients.

The Spearman correlations test was performed between the SM 42:1 plasma level and the number of coronary arteries diagnosed with atherosclerosis disease (rho = −0.356, *p* = 0.014), and the severity of the disease was measured by the Gensini Score (rho = −0.403, *p* = 0.006), as presented in Figure 2a,b.

In the next step, the possible correlations between plasma sphingomyelin levels and NLR and C-reactive protein were analyzed, and the results were not conclusive (Spearman’s rho = −0.135, *p* = 0.420 and rho = 0.204, *p* = 0.219, respectively).

We investigated the possible relationship between sphingomyelin 42:1 and co-morbidities that showed significant differences in the whole group related to dyslipidemia (*p* = 0.026) and diabetes mellitus (*p* = 0.038). Further analyses, including the relationship between sphingomyelin 42:1 and combined factors of dyslipidemia and coronary disease (*p* = 0.109) and diabetes mellitus and coronary disease, were inconclusive based on chi-square tests (*p* = 0.612).

## 3. Discussion

We present the results of sphingomyelin metabolomic profiling of coronary artery patients and control groups that identify possible differences associated with SM 42:1. The sphingomyelin 42:1 plasma concentration can be regarded as a novel biomarker for coronary artery disease prediction, including sex disparities.

The correlation between the severity of coronary artery disease and SM 42:1 has been noted in our analysis, suggesting the possible role of sphingomyelin plasma levels on atherosclerosis progression.

Sphingomyelins belong to complex sphingolipids in human cells, composed of sphingosine, fatty acids, and phosphorylcholine. They are predominantly located on cell membranes, lipoproteins, and other lipid-rich tissue structures. Their roles in growth factor receptors, supracellular matrix protein activity, and membrane permeability have been postulated [16]. The hydrolyzation of sphingomyelin can be triggered by tumor necrosis factor (TNFσ), interleukin (IL-1), gamma radiation, and interferon (IFNγ), leading to the release of ceramide via activation of sphingomyelinase. It may also regulate the activation of the isoenzyme of protein kinase C, protein phosphatase, and protein kinase [17]. The sphingolipids and their metabolites may be involved in dualistic processes regulating contrary activities [18].

Sphingomyelin’s role in neurological signal transduction is well described [19,20] and is next to regulatory participation in inflammatory processes and oxidative stress responses. In an animal study by Tchekalarova et al. [21], oxidative stress-induced sphingomyelin levels decreased in the hippocampus. The link between the lower levels of plasma sphingomyelins and coronary atherosclerosis may be explained by CAD-accompanied oxidative stress activation.

Plasma sphingolipids are erratic and mainly represent the complex of sphingomyelins, glycosphingolipids, and ceramides [22]. In the blood circulatory environment, insoluble lipids are associated with apolipoprotein, and sphingomyelins are distributed into VLDL/LDL and HDL [23]. The sphingomyelin cycle describes the chain of processes involved in sphingomyelin generation through sphingomyelin synthase (SMS), which comprises two isozymes that transfer phosphocholine of phosphatidylcholine to ceramide in mammals. Conversely, ceramide is generated from SM hydrolysis by sphingomyelinases [24]. Those by-products may trigger various reactions related to signal transduction, cell migration, and inflammatory activation [25]. The second possible explanation of our analysis results may be based on the theoretical scenario that active inflammatory cells may consume the sphingomyelin, resulting in coronary plaque formation and expansion with secondary plasma sphingomyelin decrease. On the contrary, the other by-products of the cycle, such as ceramide, exhibit physiological actions related to apoptosis and autophagy [26].

Lower plasma sphingomyelin levels can affect atherosclerotic plaque formation. The suggested increased sphingomyelin content in atherosclerotic plaques was proposed in the study by Surendran et al. [27], as its upsurge after percutaneous procedures was noted and related to the no-reflow syndrome. The third possible pathophysiological explanation of our results can be formed based on the mentioned publication of Surendran et al. Lower plasma sphingomyelin levels were observed in our study, and the Surendarn publication may explain the described phenomenon. Atherosclerotic plaque formation that requires sphingomyelins for its development results in lower plasma sphingomyelin levels; moreover, the percutaneous procedure results in plaque destruction and triggers sphingomyelin release into the circulation, as an increase in plasma sphingomyelin levels is observed in no-reflow syndrome when the atherosclerotic fragments are released into microcirculation.

Their role in apoptosis processes can explain the role of sphingomyelin in atherosclerotic plaque formation, as the mentioned pathway has been postulated in oncology. Programmed cell death was induced by sphingomyelins via activation of the Fas ligand/receptor system [28]. We suspect that one of the possible explanations for lower sphingomyelin plasma levels may be related to their role in programmed cell processes involved in atherogenesis.

In animal models, brain injury in mass spectrometry imaging resulted in lipid metabolism derangements presenting as lower sphingomyelin 42:1 levels [29]. Our analysis was performed on two groups presenting with anginal equivalent symptoms that presented natural differences between sex prevalences for coronary artery disease [30,31,32]. Relating the coronary artery disease risk to sex differences, the neutrophil-to-lymphocyte ratio and sphingomyelin 42:1 were suggestive. The sphingomyelin sex-related differences could represent one possible explanation of male prevalence in cardiovascular disease epidemiology.

Among the simple inflammatory markers from peripheral blood count analysis, the neutrophil-to-lymphocyte ratio (NLR) was distinctive for coronary artery disease [33,34,35]. This novel marker indicating inflammatory activation in atherosclerosis processes was found to be predictive of long-term prognosis [36]. Our analysis did not confirm the relationship between NLR and sphingomyelin plasma levels, suggesting a distinctive pathway for atherosclerotic plaque formation associated with SM levels. Our analysis did not identify any correlation between SM42:1 and serum C-reactive protein, one of the most common inflammatory markers used in clinical practice.

We wish to highlight that our results highlight differences in sphingomyelin plasma concentrations between coronary and non-coronary disease patients that were unrelated to inflammatory markers but may not only represent the novel pathway in atherosclerotic plaque formation but also indicate male prevalence in cardiovascular morbidity.

### Study Limitations

The study was performed on a limited number of patients, and sex differences related to coronary artery disease were corrected for results presentation. The possible novel pathway related to coronary artery disease development should be confirmed in a larger population, with possible division based on sex differences.

Our analysis was performed on patients with chronic coronary syndrome to obtain homogeneous groups. In acute coronary syndrome, the possible release of sphingomyelins into the circulation should be considered, as in previous studies; the percutaneous procedures provoked their upsurge [27].

The relationship between SM42:1 and the low- and high-density lipoprotein serum concentrations was determined without exploring their composition. Future studies on the relationship between the composition of LDL or HDL and SM42:1 should be conducted to better understand the presented phenomenon.

## 4. Materials and Methods

Comparative prospective analysis was performed between 23 (18 (72%) male) patients with a median age of 69 (63–72) years presenting with chronic coronary syndrome and confirmed epicardial disease in coronary angiography and 15 (3 (33%) male) patients with a median age of 70 (64–72) years with normal angiograms. All patients were electively admitted for internal medicine and arterial hypertension medicine due to angina-equivalent symptoms.

Patients presenting with acute coronary syndrome or presenting at least moderate valvular pathology were not included in the analysis. Patients on strict diets or presenting with chronic inflammatory conditions were excluded from the analysis.

### 4.1. Methods

Blood samples for laboratory tests were taken on admission using a routine hematology analyzer (Sysmex Europe, Norderstedt, Germany). All the lipid analyses were performed at the Medical University Central Laboratory using an Alinity Abbot analyzer (Abbot Laboratories, Abbot Park, IL, USA). Additional blood samples were obtained for metabolomic profiling.

Transthoracic echocardiography was performed by experienced echocardiographers and followed by coronary angiography performed in a planned manner in the regional reference hemodynamic center by a team of experienced interventional cardiologists. Advancement of coronary artery disease was calculated using the Gensini score [37].

Detailed sphingomyelin profiling included a triple quadrupole tandem mass spectrometry system (LC-MS/MS). The methodology is based on the commercially available kit AbsoulteIDQ p180 (Biocrates LifeSciences AG, Innsbruck, Austria). It enables quantitating a spectrum of metabolites in a single analytical run. For quantitation of all analytes, 10 µL of sample was needed. All samples were blinded and randomized prior to the study to achieve maximum reliability. Sample preparation included one common procedure for all analytes. All procedures were performed according to manufacturer specifications. In brief, the sample preparation was based on the stepwise addition of reagents to a 96-well plate. Firstly, the dissolved internal standards were pipetted onto the plate. Then, 10 µL of calibrators, quality control (QC) samples (provided by the manufacturer), and patient serum samples were pipetted directly into the wells. The plates were dried under nitrogen flow, followed by adding the derivatization solution. The plate was covered and incubated in a laboratory hood for 25 min. After this, a second drying process (1 h) was performed using nitrogen as an inert gas. Then, the extraction solution was added, and the plate was incubated on a laboratory shaker for 30 min (450 rpm). After the incubation, the mixture in each well was filtered into the capture plate (lower layer of 96-well pate) using the nitrogen pressure. The fill levels were checked, and the extracts were divided into two separate 96-well plates. The contents of both plates were diluted with the appropriate solvent according to the manufacturer’s instructions, and after sealing with a lid, both plates were placed in the autosampler.

Quantitation was performed using a 1260 Infinity HPLC (Agilent Technologies, Santa Clara, CA, USA) liquid chromatograph coupled to a 4000QTRAP mass spectrometer (SCIEX, Framingham, MA, USA) equipped with an electrospray ionization ion source. The lipid compounds were analyzed using flow injection analysis (FIA-MS/MS method) according to manufacturer specifications. The spectrometer was operated in MRM (multiple reaction monitoring) mode for the highest sensitivity and specificity. The manufacturer selected the LC-MS/MS operating parameters (including MRM transitions) and applied them during the determinations after the data had been validated by SST (system suitability tests).

### 4.2. Statistical Analysis

The Shapiro–Wilk test was used to assess the normality of data distribution. Continuous variables were presented as a median and interquartile range [Q1–Q3] and compared with the non-parametric Mann–Whitney test. Where applicable, categorical data were expressed as numbers and percentages and compared with Fisher’s exact test or Chi-square. Spearman correlation analysis was used to describe the correlation between the variables. Statistical analysis was performed using JASP software [JASP Team, Netherlands; 2023. Version 0.18.1]. *p* < 0.05 was considered statistically significant (https://jasp-stats.org/download/ accessed on 4 November 2023).

### 4.3. Bioethics Committee

The Institutional Ethics Committee of Poznan University of Medical Sciences, Poznan, Poland (protocol code 694/20 on 4 November 2020) approved the study, which was conducted according to the principles outlined in the Declaration of Helsinki.

## 5. Conclusions

Our single-center perspective analysis results indicate a possible novel role of sphingomyelin 42:1 in coronary artery disease progression. Its lower plasma level may suggest the interplay between atherosclerotic processes and an inverse relationship with coronary disease severity, suggesting the presented hypothesis. According to our preliminary report, sphingomyelin 42:1 may be considered an independent marker for CAD and not related to inflammatory reactions, as estimated by easily accessible simple peripheral blood indices such as the neutrophil-to-lymphocyte ratio. Further studies are required to confirm the presented thesis.

## Figures and Tables

**Table 1:** Demographics and clinical characteristics of analyzed groups.

Parameters	CAD Group n = 23	Control Group n = 15	*p*
Sex (m (%)/f (%))	18 (78)/5 (22)	5 (33)/10 (67)	0.007
Age (years) (median (Q1–Q3))	69 (63–72)	70 (64–72)	0.765
Height (cm) (median (Q1–Q3))	170 (164–175)	164 (162–176)	0.385
Weight (kg) (median (Q1–Q3))	79 (72–92)	80 (67–88)	0.731
BMI (kg/m^2^) (median (Q1–Q3))	29.1 (25.3–30.9)	27.9 (25.1–32.0)	1.000
Co-morbidities:			
HA (n (%))	20 (87)	12 (80)	0.587
DM (n (%))	7 (30)	4 (27)	0.820
Dyslipidemia (n (%))	19 (83)	13 (87)	0.759
COPD (n (%))	5 (22)	1 (7)	0.228
Kidney (n (%))	3 (13)	1 (7)	0.555
Nicotine (n (%))	7 (30)	2 (13)	0.240

Abbreviations: BMI—body mass index; cm—centimeters; COPD—chronic obstructive pulmonary disease; DM—diabetes mellitus; HA—arterial hypertension; kg—kilogram; n—number; m^2^—square meter.

**Table 2:** Comparison of laboratory results between disease and control groups.

Parameters	CAD Group n = 23	Control Group n = 15	*p*
Whole blood count:			
WBC (G/L) (median (Q1–Q3))	7.51 (96.40–8.72)	6.61 (5.56–7.32)	0.062
Neutrophil (G/L) (median (Q1–Q3))	5.07 (4.12–5.75)	3.88 (3.18–4.80)	0.014
Monocyte (G/L) (median (Q1–Q3))	0.45 (0.42–0.53)	0.53 (0.44–0.62)	0.155
Lymphocyte (G/L) (median (Q1–Q3))	1.53 (1.24–2.17)	1.75 (1.59–1.85)	0.395
NLR (median (Q1–Q3))	2.86 (2.45–3.95)	2.09 (1.81–2.66)	0.016
MLR (median (Q1–Q3))	0.28 (0.22–0.38)	0.30 (0.26–0.35)	0.701
Hb (mmol/L) (median (Q1–Q3))	9.20 (8.55–9.70)	8.60 (8.05–9.05)	0.073
Hct (%) (median (Q1–Q3))	44 (42–46)	41 (41–44)	0.129
MCHC (g/dL) (median (Q1–Q3))	20.99 (20.46–21.30)	20.70 (20.35–20.90)	0.189
Plt (G/L) (median (Q1–Q3))	216 (205–235)	221 (190–257)	0.929
Liver function test:			
ALT (IU/L) (median (Q1–Q3))	31 (26–42)	29 (22–34)	0.311
Kidney function test:			
Serum creatinine (mmol/L) (median (Q1–Q3))	85 (74–102)	76 (71–89)	0.220
Lipoprotein (mg/dL) (median (Q1–Q3))	0.300 (0.295–0.800)	0.130 (0.100–0.205)	0.067
Myocardial injury tests:			
CK-MB (ng/mL) (median (Q1–Q3))	14.85 (3.03–16.02)	0.74 (0.58–1.70)	<0.001
Uric acid (mg/dL) (median (Q1–Q3))	396 (380–413)	305 (266–354)	0.126
C—reactive protein (mg/L) (median (Q1–Q3))	6 (5–7)	6 (4–7)	0.439
Lipidorgam:			
Total cholesterol (mmol/L) (median (Q1–Q3))	3.83 (2.98–4.73)	4.42 (3.86–4.84)	0.252
HDL (mmol/L) (median (Q1–Q3))	1.05 (0.90–1.22)	1.56 (1.26–1.69)	0.003
LDL (mmol/L) (median (Q1–Q3))	2.45 (1.67–3.22)	2.20 (1.70–2.93)	0.843
Triglycerides (mmol/L) (median (Q1–Q3))	1.30 (1.03–1.92)	1.41 (0.93–1.83)	0.781

Abbreviations: CK-MB—creatine kinase MB; dL—deciliter; g—gram; Hb—hemoglobin; Hct—hematocrit; HDL—high-density lipoprotein; L—liter; LDL—low-density lipoprotein; NLR—neutrophil-to-lymphocyte ratio; MCHC—mean corpuscular hemoglobin concentration; mmol—millimole; MLR n—number; Plt—platelets; Q—quartiles; WBC—white blood count.

**Table 3:** Comparison of plasma sphingomyelin levels between disease and control groups.

Sphingomyelins (SM) (uM)	Whole Analyzed Group n = 38	CAD Group n = 23	Control Group n = 15	*p*
SM 33:1 (median (Q1–Q3)	5.18 (4.17–6.63)	4.9 (4.3–6.0)	6.67 (4.0–7.6)	0.521
SM 34:1 (median (Q1–Q3))	92.0 (81.4–102.5)	86.4 (81.4–96.7)	101.0 (82.3–120.0)	0.078
SM 34:2 (median (Q1–Q3))	13.7 (11.7–16.0)	12.8 (11.3–14.8)	15.5 (12.5–18.3)	0.054
SM 35:1 (median (Q1–Q3))	3.3 (2.5–4.1)	3.2 (2.6–3.8)	4.0 (2.5–4.5)	0.473
SM 36:1 (median (Q1–Q3))	22.5 (20.2–24.6)	22.3 (20.1–23.9)	23.8 (21.6–26.6)	0.151
SM 36:2 (median (Q1–Q3))	10.95 (8.82–11.88)	10.4 (8.6–11.5)	11.7 (10.0–13.2)	0.091
SM 38:3 (median (Q1–Q3))	0.37 (0.27–0.45)	0.4 (0.3–0.4)	0.4 (0.3–0.5)	0.375
SM 40:4 (median (Q1–Q3))	1.285 (1.060–1.548)	1.2 (1.1–1.3)	1.5 (1.2–1.8)	0.091
SM 41:1 (median (Q1–Q3))	10.55 (8.86–13.38)	10.4 (8.8–10.9)	13.5 (9.3–15.7)	0.113
SM 41:2 (median (Q1–Q3))	10.2 (8.2–12.5)	10.1 (8.3–10.7)	12.7 (7.9–15.0)	0.215
SM 42:1 (median (Q1–Q3))	18.15 (15.53–21.08)	16.2 (14.2–19.1)	20.8 (18.9–21.7)	0.044 *
SM 42:2 (median (Q1–Q3))	49.9(44.1–57.0)	47.0 (43.7–55.9)	55.1 (45.7–62.1)	0.189
SM 43:1 (median (Q1–Q3))	1.19 (1.05–1.44)	1.1 (1.1–1.3)	1.4 (1.0–1.6)	0.199
SM 44:1 (median (Q1–Q3))	0.175 (0.151–0.218)	0.17 (0.15–0.22)	0.19 (0.15–0.21)	0.765
SM 44:2 (median (Q1–Q3))	0.49 (0.43–0.65)	0.46 (0.42–0.62)	0.54 (0.46–0.68)	0.483

Abbreviations: SM 33:1—SM(d18:0/14:1(9Z)(OH)) chemical assignment of Hydroxysphingomyeline C14:1; SM 34:1—SM(d18:1/16:0) consists of a sphingosine backbone and a palmitic acid chain; SM 34:2—SM(d18:2/16:0); SM 35:1—SM(d18:0/16:1(9Z)(OH)) chemical assignment of Hydroxysphingomyeline C16:1; SM 36:1—SM(d18:1/18:0) consists of a sphingosine backbone and a stearic acid chain; SM 36:2—SM(d18:1/18:1(11Z)) or SM(d18:1/18:1(9Z)) that consists of a sphingosine backbone and an oleic acid chain; SM 38:3—SM(d18:2/18:1); SM 40:4—SM(d18:2/22:2); SM 41:1—SM(d17:1/24:0) consists of a heptadecasphingosine backbone and a lignoceric acid chain/SM(d18:1/23:0) consists of a sphingosine backbone and a tricosanoic acid chain/SM(d18:0/22:1(13Z)(OH)) chemical assignment of Hydroxysphingomyeline C22:1; SM 41:2—SM(d17:1/24:1(15Z)) consists of a heptadecasphingosine backbone and a nervonic acid chain/SM(d18:0/22:2(13Z,16Z)(OH)) chemical assignment of Hydroxysphingomyeline C22:2; SM 42:1—SM(d18:1/24:0) consists of a sphingosine backbone and a lignoceric acid chain; SM 42:2—SM(d18:1/24:1(15Z)) consists of a sphingosine backbone and a nervonic acid chain; SM 43:1—SM(d18:0/24:1(15Z)(OH)) chemical assignment of Hydroxysphingomyeline C24:1; SM 44:1—SM(d18:1/26:0) consists of a sphingosine backbone and a cerotic acid chain; SM 44:2—SM(d18:1/26:1(17Z). *—statistically significant.

**Table 4:** Fisher’s exact test for CAD in male patients.

Parameters	CAD Prediction, Including Male Sex		
	Log Odds Ratio	95% Confidence Interval	*p*
SM 42:1	1.97	1.62–2.32	<0.001
Neutrophil count	1.99	1.25–2.77	<0.001
NLR	1.72	0.78–2.71	<0.001

Abbreviations: NLR–neutrophil-to-lymphocyte ratio.

## Data Availability

Data supporting the reported results can be obtained after presenting a reasonable request by contacting the corresponding authors via e-mail for three years after publication. The restrictions are related to privacy and ethical motivations.
